# Prevalence and correlates of cigarette smoking among Dulong adults in China: A cross-sectional survey in 2020

**DOI:** 10.3389/fpubh.2022.973583

**Published:** 2022-10-13

**Authors:** Ying Shao, Shun Zha, Mingfang Qin, Qiuyan Zhu, Xiliang Yang, Cangjiang Yang, Xinlin Wang, Yanli Zhang, Weimei Yang, Kunhua Zhou, Yanmei Li, Xian Tang, Qiuli Yu

**Affiliations:** ^1^Division for Prevention and Control of Chronic Non-communicable Disease, Yunnan Center for Disease Control and Prevention, Kunming, China; ^2^Executive Office, Yunnan Institute of Endemic Disease Control, Dali, China; ^3^Department for Prevention and Control of Chronic Non-communicable Disease, Nujiang Prefecture Center for Disease Control and Prevention, Lushui, China; ^4^Executive Office, Gonshan County Center for Disease Control and Prevention, Gongshan, China

**Keywords:** cigarette smoking, prevalence, risk factors, minority, Dulong adult

## Abstract

**Background:**

The Dulong people are one of the minorities in China with the lowest population. In recent years, the lifestyle of the Dulong people has also changed drastically due to income growth and urbanization. This study aims to identify cigarette smoking prevalence and potential risk factors among Dulong adults in China.

**Methods:**

This study was conducted among 1,018 adults based on the Dulong Health Status Investigation and Evaluation (DHSIE) in Gongshan Dulong and Nu Autonomous County of Yunnan province, Southwest China. A cross-sectional design and face-to-face questionnaire were used to collect cigarette smoking habits and demographic information. Data were weighted by post-stratification weights according to the age and gender composition of Dulong resident. We also analyzed univariate and multivariate unconditional logistic regression to explore current smoking correlates.

**Results:**

The weighted prevalence of ever-smoking, currently smoking, and formerly smoking among Dulong adults is 31.3, 27.7, and 3.6%, respectively. The prevalence of ever-smoking and currently smoking among male participants (57.0 and 50.6%) is much higher than that of female participants (4.0 and 3.4%). Nearly 60% of ever-smokers and current smokers smoked more than 20 cigarettes per day, which are higher than former smokers (35.2%). Among current smokers, 33.1% relapsed, and 28.3% intend to quit smoking. By adjusting for potential confounding variables, multiple logistic regression analysis indicated that male participants (OR = 48.982, 95% CI: 25.026–95.869) and current drinkers (OR = 4.450, 95% CI: 2.556–7.746) are more likely to be current smokers. On the contrary, current smokers are also more likely to be exposed to secondhand smoke (OR = 4.269, 95% CI: 2.330–7.820) and have a higher risk of chronic respiratory disease (OR = 4.955, 95% CI: 1.669–14.706).

**Conclusion:**

Cigarette smoking is highly prevalent among the Dulong people in Southwest China. An appropriate and effective tobacco control strategy is an urgent need for this population.

## Introduction

Smoking leads to various diseases and is the leading cause of premature death and disability (1). Tobacco use causes more than 6 million deaths yearly (2), mainly in low-income and lower-middle-income countries (3). The contribution of smoking to the overall disease burden has been increasing since 1990 (1), whereby billions of dollars in healthcare costs are lost every year due to smoking in the world (4). Tobacco production and consumption in China are among the highest in the world (5), as just in 2019, China produced about 2,364 billion cigarettes (6), accounting for about 40% of the global total (7). According to the 2010 global burden of disease study in China (8), smoking is the second leading risk factor for disability-adjusted life years (DALYs) and the number of deaths. DALYs attributable to smoking were 30 million person-years, accounting for 9.5% of the total disease burden attributed to behavioral risk factors. The disease burden caused by smoking was mainly cardiovascular and circulatory diseases (63.5%), followed by cancer (18.7%) and chronic respiratory diseases (13.1%) (8). Compared with 2010, smoking rose to the leading risk factor for DALYs in 2017, and both DALYs and deaths attributed to smoking have increased (9). It was estimated that the smoking rate of Chinese adults was 27.3% (51.8% men and 2.3% women) in 2013–2014 (10). Moreover, research reveals that smoking is a significant public health issue in rural areas of Central and Western China (11). The prevalence of smoking in rural areas is more common among ethnic minorities and people with poor socioeconomic status (12, 13). According to previous studies in other Chinese provinces (14–17), lower educational attainment, alcohol consumption, and occupation were the major factors related to smoking.

China is multi-ethnical, with the Han as the majority and 55 other ethnic minorities. The Dulong is one of the scarcely populated minorities in China, and the ethnic group with the smallest population in Yunnan Province, compared to other ethnic groups. According to the Sixth National Census (18), the population of the Dulong people is <7,000. Most of them live in Gongshan Dulong and Nu Autonomous County of Nujiang Prefecture in Yunnan Province, the most remote, impoverished, and inaccessible part of China. Due to the harsh climate, the economic and social development of the region is relatively backward, and the Dulong residents have a poor ability to obtain medical care services (19). In recent years, with economic development and urbanization in China, the disease burden attributable to income growth and urbanization has increased (20), and the lifestyle of the Dulong people has also changed with it. However, unhealthy behaviors such as smoking, alcohol drinking, and lack of physical activity may still be prevalent. Medical accessibility and affordability were positively associated with healthy life expectancy in China (21). Therefore, the harm caused by smoking poses a more severe threat to the Dulong than other minor ethnicities. Furthermore, Yunnan Province is a significant tobacco-growing and cigarette manufacturing area in China. Similar to the results of a previous study in China (22), tobacco control in Yunnan is facing an economic and public health crossroads: although smoking harms people's health, restraining smoking threatens social stability and government revenue; consequently, controlling tobacco consumption is challenging, especially in rural areas inhabited by ethnic minorities.

Nonetheless, a comprehensive report on the chronic disease and risk factors among the Dulong in this area has yet to be conducted. To fill this gap, we report relevant data based on the Dulong Health Status Investigation and Evaluation (DHSIE), to identify the prevalence, potential factors, and disease-related smoking among the Dulong people. The findings would serve as a keynote in providing information to policymakers and public health scholars to further tackle the abuse of tobacco consumption.

## Methods

### Survey site and participants

The study was designed as a cross-sectional survey based on the Dulong Health Status Investigation and Evaluation (DHSIE) and with the support of the Health Commission of Yunnan Province. The survey and interviews were designed and conducted from 2020 to 2021. It was conducted in the Dulongjiang township in Gongshan Dulong and Nu Autonomous County of Nujiang Prefecture in Yunnan Province, Southwest China. The reason for choosing this township is that Gongshan County has the largest and most concentrated population of Dulong people in China, where over 90% of Dulong people in Gongshan County live in this township.

Participants who lived in six administrative villages (Kongdang, Dizhengdang, Longyuan, Xianjiudang, Bapo, and Maku) were selected through multi-stage cluster sampling, from 20 August to 6 September 2020. In the first step, two village groups were randomly selected from each administrative village. Next, 30 households were randomly selected from each village group in the second step. Finally, all 1,065 eligible members of the households were included in the survey. Due to refusal to investigate or temporary absence, 1,018 participants aged 18 and over were included in this survey, and the response rate was 95.6%. The inclusion criteria for our study were as follows: of Dulong ethnicity, over 18 years old, have lived in the local area for more than 6 months in the past year, and have no cognitive impairment. The village cadres would call or enter the household to inform the eligible respondents of the investigation's purpose, content, time, and place. The written consent form was obtained when the field survey was conducted.

The sample size calculation of the DHSIE was based on the following formula:


$$\begin{array}{l} N=\frac{u_{\alpha }^{2}P\left(1-P\right)}{d^{2}} \end{array}$$

α is the significance level, *u*_α_ is equal to 1.96, *P* is the prevalence of hypertension in China in 2013 (27.8%) (23), and *d* is the error tolerance, which can be estimated as relative error × *p*; the relative error is 0.10. The estimated minimum sample size is 998.

### Data collection

The DHSIE included questionnaire interviews, physical measurements, and laboratory examinations. A face-to-face questionnaire was used to collect demographic characteristics, smoking status, passive smoking, knowledge of smoking and passive smoking hazard, alcohol drinking, history of chronic respiratory diseases, and self-assessment of health status. The questions about smoking status included currently smoking, former smoking, frequency, and amount of cigarettes smoked per day by current smokers. It is not common for the Dulong to smoke substances other than tobacco, so smoking in this survey refers only to smoking cigarettes.

The field survey was conducted at the township health center or village clinics. All interviewers were medical staff who went through training according to the protocol. Clinical and epidemiological experts discussed and revised the protocol and questionnaire multiple times. A small-scale pre-survey was conducted on the general population to improve the protocol and questionnaire.

### Measures

#### Outcome variables

The tobacco use-related information included smoking patterns, frequency, amount, and smoking cessation; all were self-reported. Based on the smoking patterns, participants were categorized into three smoking groups: current, former, and never smoked. Current and former smokers were classified as ever-smokers. For ever-smokers, information on the frequency of smoking, daily cigarette consumption, relapse smoking (for the current smoker), intend to quit smoking (for the current smoker), reasons to quit smoking, and duration of smoking cessation (for former smokers) was collected.

Current smokers were those who have smoked in the past year. Former smokers were those who regularly smoked in the past but have not for at least 1 year preceding the survey. Heavy smoker was defined as someone who smokes more than 20 sticks daily (24, 25). Passive smoking is defined as exposure to secondhand smoke at least once a week.

#### Potential factors

Education is classified into three categories: low, medium, and high, which refers to primary school or lower, middle or high school, and college or higher, respectively. The occupation was divided into four groups: (1) farmer, defined as being engaged in agriculture, forestry, animal husbandry, and fishery for a living; (2) official staff, which refers to people working in governmental or professional technical organizations; (3) housework, for instance homemakers; (4) others, people who could not be categorized to the groups above, such as those unemployed, soldiers, students, business people and service staff, workers, etc.

To measure one's knowledge about smoking hazards, the question “As far as you know, will smoking cause the following diseases: stroke, heart attack, and lung cancer?” was used. Participants were also asked whether secondhand smoke will cause heart disease or lung cancer in adults and other related respiratory symptoms in children to assess knowledge about passive smoking hazards.

Current drinkers were those who have drunk alcohol in the past year and were still drinking in the past month (26). According to the system review recommended by WHO, we classify dangerous and harmful drinking as heavy drinking, which refers to an average daily alcohol intake of ≥41 g for men and ≥21 g for women (27).

In this study, medical institutions defined participants who had been diagnosed with chronic obstructive pulmonary disease (COPD), chronic bronchitis, or emphysema before the investigation as having a self-report history of chronic respiratory diseases.

### Statistical analysis

To reduce the sample's structure deviation from the Dulong population, data were weighted by post-stratification weights (28), according to the age and gender composition of Dulong residents. Numerical variables were represented as mean ± standard deviation (SD), and categorical variables were presented as number percentages. We also analyzed univariate and multivariate unconditional logistic regression to explore current smoking correlates. The stepwise selection of variables in multivariate logistic regression was not used, but all relevant variables were included in the model. A confidence value of *P* < 0.05 was considered statistically significant. All statistical analyses were performed with SPSS 18.0 for Windows (SPSS Inc., Chicago).

### Ethics statement

The study protocol followed the Helsinki Declaration and was approved by the Institutional Review Board (IRB) of the Yunnan Center for Disease Control and Prevention (reference number 2020-11). The approval certificate is available as an Appendix.

The purpose and nature of the study were explained to eligible participants who signed a written consent form indicating their agreement to participate. The informed consent describes the investigation content, confidentiality commitment, rights, and risks of the respondents of this study. If the respondents participate in this study, a questionnaire survey, physical examination, and blood and urine samples for laboratory examination will be conducted on-site.

## Results

### Demographic characteristics and smoking prevalence

**Figure 1:**
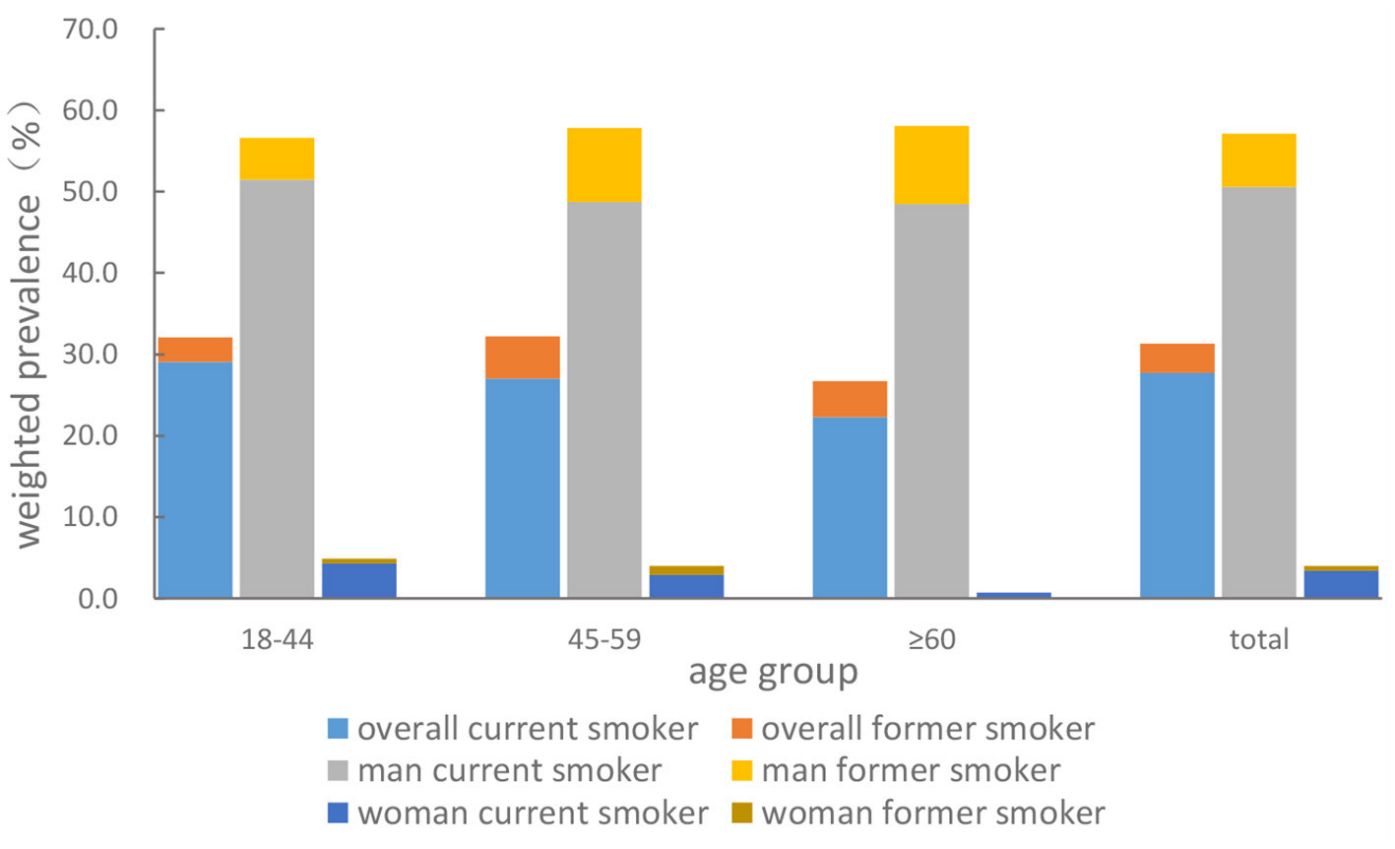
Weighted smoking prevalence of ever-smoking, currently smoking, and former smoking among Dulong people aged 18 and above in 2020.

After post-stratification weighting, among the 1,018 participants, the mean age was 41.1 ± 0.6 years old, 51.5% were male participants, 51.8% had low education, 43.8% had medium education, 16.6% were never married, and 42.7% were farmers (Table 1). The weighted prevalence of ever-smoking, currently smoking, and former smoking is 31.3, 27.7, and 3.6%, respectively (Table 2, Figure 1). We found that the prevalence of ever-smoking in male participants (57.0%) is much higher than that in female participants (4.0%). Participants with medium and high education levels had a high prevalence of ever-smoking (36.7 and 36.3%) than participants with a low education level (26.3%). Compared with married, divorced, or widowed participants, never married subjects had a higher prevalence of ever-smoking in both sexes (Table 2). However, there was no significant difference in the prevalence of ever-smoking among different age groups (Table 2, Figure 1).

**Table 1:** Demographic characteristics of the participants.

**Variables**	**Overall**			**Man**			**Woman**		
	***N***	**Constituent ratio**	**Weighted constituent ratio**	***N***	**Constituent ratio**	**Weighted constituent ratio**	***N***	**Constituent ratio**	**Weighted constituent ratio**
**Total**	1,018	100.0	100.0	385	100.0	100.0	633	100.0	100.0
**Age**
18–44	610	59.9	65.3	240	62.3	66.7	370	58.5	63.9
45–59	263	25.8	19.9	89	23.1	20.3	174	27.5	19.5
≥60	145	14.2	14.8	56	14.5	13.0	89	14.1	16.6
**Education**
Low	583	57.3	51.8	201	52.2	47.0	382	60.3	56.9
Medium	393	38.6	43.8	164	42.6	47.8	229	36.2	39.6
High	42	4.1	4.4	20	5.2	5.2	22	3.5	3.5
**Marital status**
Never married	117	11.5	16.6	78	20.3	25.0	39	6.2	7.5
Married	837	82.2	77.4	291	75.6	71.2	546	86.3	84.1
Divorced or widowed	64	6.3	6.0	16	4.2	3.9	48	7.6	8.4
**Occupation**
Farmer	430	42.2	42.7	180	46.8	45.5	250	39.5	39.8
Official staff	42	4.1	4.3	20	5.2	5.0	22	3.5	3.5
Housework	366	36.0	34.2	105	27.3	28.0	261	41.2	40.8
Others	180	17.7	18.8	80	20.8	21.5	100	15.8	15.9
**Current drinker**
Yes	472	46.4	48.2	192	50.0	50.8	280	44.2	45.4
No	546	53.6	51.8	193	50.0	49.2	353	55.8	54.6
**Heavy drinking**
Yes	123	12.1	12.0	51	13.3	13.8	72	11.3	10.0
No	895	87.9	88.0	334	86.7	86.2	561	88.7	90.0
**Passive smoking**
Yes	669	65.7	65.5	246	63.8	64.3	423	66.9	66.9
No	349	34.3	34.5	139	36.2	35.7	210	33.1	33.1
**Awareness of smoking hazards**									
Yes	512	50.3	50.4	205	53.3	53.8	307	48.5	46.9
No	506	49.7	49.6	180	46.7	46.2	326	51.5	53.1
**Awareness of passive smoking hazards**									
Yes	431	42.3	42.4	170	44.2	45.3	261	41.2	39.3
No	587	57.7	57.6	215	55.8	54.7	372	58.8	60.7
**Self-report history of chronic respiratory diseases**									
Yes	35	3.4	3.5	21	5.4	4.6	14	2.2	2.4
No	983	96.6	96.5	364	94.6	95.4	619	97.8	97.6
**Self-assessment of health status**									
Good	377	37.0	36.1	149	38.8	41.7	228	36.0	30.2
Average	502	49.3	50.6	189	49.0	46.8	313	49.5	54.6
Bad	139	13.7	13.3	47	12.3	11.5	92	14.5	15.2

**Table 2:** Prevalence of cigarette smoking of the participants by demographic characteristic.

**Variables**	**Overall**				**Man**				**Woman**			
	**Never smokers**		**Ever smoker**		**Never smokers**		**Ever smoker**		**Never smokers**		**Ever smoker**	
	***N***	**%^a^**	***N***	**%^a^**	***N***	**%^a^**	***N***	**%^a^**	***N***	**%^a^**	***N***	**%^a^**
**Total**	774	68.7	244	31.3	167	43.0	218	57.0	607	96.0	26	4.0
**Age**												
18–44	458	67.9	152	32.1	106	43.4	134	56.6	352	95.1	18	4.9
45–59	205	67.8	58	32.2	38	42.2	51	57.8	167	96.0	7	4.0
≥60	111	73.3	34	26.7	23	41.9	33	58.1	88	99.3	1	0.7
*P*-value for trend	0.489				0.969				0.080			
**Education**												
Low	466	73.7	117	26.3	96	46.9	105	53.1	370	97.2	12	2.8
Medium	278	63.3	115	36.7	63	39.3	101	60.7	215	94.0	14	6.0
High	30	63.7	244	36.3	8	41.0	12	59.0	22	100.0	0	0.0
*P*-value for trend	0.008				0.395				0.093			
**Marital status**												
Never married	59	42.7	58	57.3	25	30.9	32	69.1	34	85.1	5	14.9
Married	661	73.1	176	26.9	136	47.5	178	52.5	526	96.7	20	3.3
Divorced or widowed	54	78.4	10	21.6	6	38.0	8	62.0	47	98.6	1	1.4
*P*-value for difference	<0.001				0.032				0.001			
**Occupation**												
Farmer	325	69.2	105	30.8	81	45.7	99	54.3	244	97.7	7	2.3
Official staff	30	65.8	12	34.2	8	43.412		56.6	21	100.0	0	0.0
Housework	290	71.8	76	28.2	43	40.1	62	59.9	248	94.9	13	5.1
Others	129	63.0	51	37.0	35	41.5	45	58.5	94	93.8	6	6.2
*P*-value for difference	0.306				0.827				0.210			

^a^The weighted prevalence, stratified by demographic characteristics.

### Smoking patterns

The proportion of daily smokers among ever-smokers, current smokers, and former smokers is 90.1, 93.9, and 85.1%, respectively, and the smoking patterns of participants can be seen in Table 3. Among ever-smokers and current smokers, nearly 60% smoked more than 20 cigarettes per day, higher than former smokers (35.2%). Among current smokers, 33.1% relapsed, and 28.3% intend to quit smoking. Of former smokers, 26.0% had stopped smoking for <2 years (Table 2). The reasons for quitting smoking among former and current smokers who were willing to quit included 39.9% of former smokers quit smoking to prevent diseases, 28.2% for other reasons, 26.3% for illness, and 5.6% for economic burden (Table 3). Among current smokers willing to quit smoking, over 60% want to quit to prevent illness, and 15.3% are due to current health conditions/illnesses. Regardless of a doctor's advice to quit smoking, former or current smokers, who are willing to quit, did not choose to do so considering the advice received.

**Table 3:** Smoking patterns of the participants, stratified by smoking status.

	**Ever smoker (*****n*** = **244)**		**Current smoker (*****n*** = **215)**		**Former smoker (*****n*** = **29)**	
	***N***	**%^a^**	***N***	**%^a^**	***N***	**%^a^**
**Frequency of smoking**						
Daily	225	90.1	201	93.9	24	85.1
Less than daily	19	9.9	14	6.1	5	14.9
*P*-value for difference						0.159
**Average number of cigarettes per day**						
≥20	130	56.8	121	57.2	9	35.2
< 20	114	43.2	94	42.8	20	65.8
*P*-value for difference						0.021
**Relapse**						
Yes	–	–	69	33.1	–	–
No	–	–	146	66.9	–	–
**Intend to quit smoking**						
Yes	–	–	57	28.3	–	–
No	–	–	124	56.3	–	–
Uncertain	–	–	34	15.4	–	–
**Reasons for quitting smoking**						
Illness	17	16.3	9	15.3	8	26.3
Prevent illness	46	54.1	36	60.1	10	39.9
Financial burden	5	6.4	3	7.1	2	5.6
Family members objected	3	4.4	3	6.4	0	0.0
Doctor's advice	0	0.0	0	0.0	0	0.0
Others	15	16.3	6	11.1	9	28.2
*P*-value for difference						0.072
**Duration of cessation of smoking (year)**						
<2	–	–	–	–	6	26.0
2–9	–	–	–	–	8	25.9
5–9	–	–	–	–	4	15.3
≥10	–	–	–	–	11	32.8

^a^Weighted constituent ratio.

### Prevalence and correlates of current smoking

Detailed information about current smoking among the participants can be seen in Table 4. By adjusting for potential confounding variables, multiple logistic regression analysis indicated that current smoking is significantly associated with sex, current alcohol drinking, passive smoking, and self-reported history of chronic respiratory diseases. Male participants (OR = 48.982, 95% CI: 25.026–95.869) and current drinkers (OR = 4.450, 95% CI: 2.556–7.746) were more likely to be current smokers as well. Current smokers were also more likely to be exposed to secondhand smoke (OR = 4.269, 95% CI: 2.330–7.820) and have a higher risk of chronic respiratory disease (OR = 4.955, 95% CI: 1.669–14.706).

**Table 4:** Prevalence and correlates of current smoking among the participants.

	***N***	**Current smoking prevalence**		**Univariate**		**Multivariable**	
		***N***	**%^**a**^**	**OR (95% CI)**	***P***	**OR (95% CI)**	***P***
**Total**	1,018	215	27.7	–	–	–	
**Age**							
18–44	610	138	29.1	1.428 (0.883–2.309)	0.147	1.035 (0.503–2.132)	0.925
45–59	263	48	27.0	1.283 (0.745–2.211)	0.368	0.683 (0.326–1.429)	0.310
≥60	145	29	22.3	1.000		1.000	
**Sex**						
Male	385	193	50.5	29.016 (17.888–47.064)	<0.001	48.982 (25.026–95.869)	<0.001
Female	633	22	3.4	1.000		1.000	
**Education**							
Low	583	103	23.2	0.659 (0.313–1.388)	0.272	1.232 (0.383–3.960)	0.726
medium	393	101	32.6	1.053 (0.497–2.229)	0.893	1.515 (0.500–4.591)	0.463
High	42	11	31.4	1.000		1.000	
**Marital status**							
Never married	117	52	52.1	4.194 (1.801–9.765)	0.001	1.975 (0.711–5.487)	0.191
Married	837	153	23.4	1.177 (0.544–2.547)	0.679	1.053 (0.484–2.293)	0.895
Divorced or widowed	64	9	20.6	1.000		1.000	
**Occupation**							
Farmer	431	91	26.9	0.716 (0.467–1.099)	0.126	0.720 (0.368–1.406)	0.335
Official staff	41	10	28.8	0.787 (0.348–1.781)	0.565	0.438 (0.131–1.466)	0.180
Housework	365	66	24.9	0.645 (0.411–1.014)	0.057	0.914 (0.447–1.872)	0.806
Other	181	47	33.9	1.000		1.000	
**Current drinker**							
Yes	472	152	41.0	3.825 (2.693–5.435)	<0.001	4.450 (2.556–7.746)	<0.001
No	546	63	15.4	1.000		1.000	
**Heavy drinking**							
Yes	123	46	49.4	2.946 (1.875–4.628)	<0.001	1.708 (0.797–3.660)	0.168
No	895	169	24.9	1.000		1.000	
**Passive smoking**							
Yes	669	174	35.7	3.207 (2.166–4.748)	<0.001	4.269 (2.330–7.820)	<0.001
No	349	41	14.8	1.000		1.000	
**Awareness of smoking hazards**							
Yes	512	112	27.6	0.987 (0.718–1.357)	0.935	0.664 (0.327–1.348	0.257
No	506	103	27.9	1.000		1.000	
**Awareness of passive smoking hazards**							
Yes	431	98	29.5	1.168 (0.848–1.609)	0.341	1.506 (0.747–3.033)	0.252
No	587	117	26.4	1.000		1.000	
**Self-report history of chronic respiratory diseases**							
Yes	35	14	47.1	2.362 (1.115–5.001)	0.025	4.955 (1.669–14.706)	0.004
No	983	201	27.4	1.000		1.000	
**Self-assessment of health status**							
Good	377	80	30.7	1.421 (0.848–2.380)	0.182	0.459 (0.200–1.053)	0.605
Average	502	108	26.7	1.167 (0.710–1.918)	0.543	0.754 (0.352–1.614)	0.467
Bad	139	27	23.7	1.000		1.000	

^a^The weighted prevalence.

## Discussion

By multi-stage sampling, we collected samples of Dulong adult participants living in Yunnan Province, Southwest China. The present study revealed a high smoking prevalence and explored the pattern, influencing factors, and related diseases. These factors mentioned above and data may provide helpful evidence for policymakers to initiate more targeted and effective interventions for tobacco control.

The Framework Convention on Tobacco Control (FCTC) has played a beneficial role in tobacco control (29). However, the effect of tobacco control varies within the socioeconomic and development status of a country (2). The prevalence of tobacco usage and its associated morbidity and mortality risk mainly occurs in low- and middle-income countries (28). In China, regardless of the various measures and policies for tobacco control adopted by the government (30), the prevalence of smoking remains high. Thus, a nationally representative survey, by the China Chronic Disease and Risk Factor Surveillance (CCDRFS), showed that 51.8% of male participants and 2.3% of female participants were current smokers as of 2014 (7). In 2015, the percentage of daily smoking among Chinese adults was 37.5% for men and 2.2% for women (2). In our study, the weighted prevalence of current smoking among Dulong adults is close to that of the national survey of China (27.3%) (10). For male participants, the prevalence was lower than that of the general male population, but for female participants, the prevalence was higher than that of the general female population. Compared with other ethnic minorities in China, the prevalence of current smoking in Dulong adults was higher than that of the Hui (21.0%) (31), Hazakh (29.6%) (32), and Uighur (11.8%) (32), but lower than Naxi (31.1%) (33), Dai (41.2%) (34), and Jingpo (39.2%) (34).

Due to the higher prevalence in female populations, urgent intervention is required as the Dulong residents are associated with a variety of chronic diseases, and coupled with poor medical conditions and the inaccessibility to obtain medical attention, it is of utmost importance to carry out controlled tobacco intervention and to increase investment in health expenses (35) to prevent the smoking of cigarettes among the Dulong population.

Among current smokers, the proportion of daily smokers was close to that in Yunnan Province (93.8%) (36), but higher than the national survey in 2010 (37). The proportion of heavy smokers was higher than that in the national survey in 2005 (44.9%) (38). Pierce et al. reported that the proportion of heavy smokers among current smokers was 23 and 40% in California and in the remaining United States, respectively, in 2007 (17). These results indicated that the tobacco dependence of the Dulong was more severe than that of the whole country and developed regions abroad. Unfortunately, only 3.0% of participants have successfully quit smoking, almost a third of current smokers have relapsed, and fewer than one-third of current smokers intend to quit. The reasons for quitting smoking of current and former smokers were mainly to prevent illness or due to current illness. The financial burden has little effect on smoking cessation; notably, doctors' advice had no effect. However, previous studies have found that the most influential factors in smoking cessation are poor health or being diagnosed with chronic disease (39, 40). A survey in China has found that doctors in primary medical institutions are less likely to advise patients to quit smoking than those in high-level medical institutions (41). A likely cause of doctors' advice falling on deaf ears is the lack of care and innate stubbornness, which may result from a lack of willingness for the Dulong to quit smoking. In addition, the proportion of former smokers who smoked more than 20 cigarettes daily is lower than that of current smokers. It is easy to understand that people, who consume more cigarettes per day, find it difficult to quit smoking successfully, and previous studies show similar findings (39, 42).

When the correlation of current smoking was examined further, we found more male participants than female participants. The results indicate the consistency with the situation in various parts of China (10, 37) and other developing countries (43). In China, women are usually not encouraged to smoke, as smoking is predominantly a male phenomenon (44). However, smoking was more common in female participants among the Dulong than in the rest of the country (10). A reason for this may be related to the unique customs of ethnic minorities in Yunnan Province that discriminate less against women smoking (45). Passive smoking was common among current smokers. The activity of the Dulong smokers, who often gathered together to smoke, would be the likely reason for passive smoke. Therefore, group intervention measures can be taken in tobacco control intervention. Furthermore, the prevalence of current smokers was higher among those with self-reported respiratory disease history. In this study, smoking was more prevalent among current drinkers. Our results align with previous research, which found that heavy smoking was common among current drinkers who might also be likely to engage in risky behaviors (26). We can confirm the conclusion of previous research that substance-dependent behavior often coexists, whereas regular smokers are often co-dependent on alcohol (46). Thus, comprehensive control of tobacco and alcohol use is an effective way to reduce the risk of related diseases. However, these correlations still need to be confirmed further by longitudinal studies. It is widely known that smoking is a significant risk factor for chronic respiratory diseases. The present study revealed a high prevalence in people suffering from chronic respiratory diseases and suggests that smoking cessation intervention for Dulong patients with chronic respiratory diseases must be strengthened. Moreover, treating chronic respiratory diseases should be gradually included as an essential public health services. Therefore, effective smoking cessation consultation, intervention, and treatment for patients with chronic respiratory diseases should be provided in local medical facilities accessible to the Dulong community and others.

Compared with ethnic minorities in other countries, the prevalence of smoking among Dulong adults is higher than that of Caucasians (22.0%), African Americans (21.3%), Latino (15.8%), and Asian Americans (9.9%), but lower than that of Indigenous Americans/Alaskan Native (32.4%) in the United States (47). According to the United States with experience in tobacco control for ethnic minorities, behavioral counseling and pharmacotherapy are effective ways to aid smoking cessation (48). These previous studies highlight the importance of various effective strategies to overcome the barriers to ethnic minority smokers' utilization of evidence-based tobacco treatments, particularly pharmacotherapy. Personal beliefs, views toward doctors, and lack of knowledge are essential determinants of the use of tobacco treatments among ethnic minority smokers (13). Hence, adequate measures must be taken to improve the current severity of high smoking prevalence among Dulong adults. We should consider implementing the Framework Convention on Tobacco Control (FCTC) and Health China 2030 strategy. Such strategies include banning smoking indoors, in medical institutions, in schools (49), and in workplaces (50). Moreover, strengthening the supervision and law enforcement of tobacco advertising would control the smoker populace (5), hoping to serve them proper summons when they have committed a public misdemeanor. Public gatherings during various ethnic festivals can be used to educate the public about tobacco control, especially during World No Tobacco Day. Thus, the implications of tobacco exposure can educate residents to fully understand the severe harm of smoking and secondhand smoke exposure (51). In hindsight, law enforcement and public health officials could gradually establish and improve the smoking cessation service system. For example, opening smoking cessation clinics to provide consultation and pharmacotherapy in Dulongjiang Township Health Center would potentially quell the spread of smoking among the populace.

This study is the first community-based study of smoking among Dulong adults in China. The prevalence and correlation factors of smoking and smoking patterns of Dulong adults are revealed in the present survey. However, there are some limitations to this study. First, our study was cross-sectional in design, rather than longitudinal, which may have made the relationship between outcome and exposure more tentative. Second, all the information collected is self-reported by the respondents, which may be subject to recall bias. Third, previous studies have shown that economic income is related to smoking, and income may also be related to occupation and education. In this survey, the vast majority of respondents were unclear about or refused to provide their annual income, so this variable is not included in the analysis.

Nonetheless, this study provides important essential enlightenment for tobacco control and prevention of chronic diseases caused by tobacco among Dulong people in Southwest China. Since most of the Dulong nationality live in areas with poor medical access, it is imperative to intervene in risk factors as early as possible to avoid the occurrence of chronic diseases, which is consistent with the goal of the national health poverty alleviation and the Healthy China 2030 programs (52). The appropriate tobacco control strategy to effectively change the unhealthy lifestyle of ethnic minorities is worth of further studying.

## Conclusion

The findings of this study revealed that cigarette smoking is highly prevalent among Dulong people in Southwest China, and evidence of smoking patterns is also provided. Factors such as alcohol drinking are found to be associated with current smoking. The high current smoking prevalence in people with chronic respiratory diseases shows a tremendous challenge for tobacco control for the Dulong. Since most of the Dulong people live in areas with poor medical access, a targeted tobacco cessation strategy should be initiated early.

## Data availability statement

The original contributions presented in the study are included in the article/supplementary material. The datasets presented in this study can be found in online repositories. The names of the repository can be found at: https://dataverse.harvard.edu/dataset.xhtml?persistentId=doi:10.7910/DVN/FOWDSK. Further inquiries can be directed to the corresponding author.

## Ethics statement

The studies involving human participants were reviewed and approved by Institutional Review Board (IRB) of Yunnan Center for Disease Control and Prevention. The patients/participants provided their written informed consent to participate in this study.

## Author contributions

YS conceived the study, analyzed the data, and wrote the manuscript. SZ and MQ guided the data analysis procedure and reviewed the manuscript. QZ, XY, CY, XW, YZ, WY, KZ, YL, XT, and QY contributed to the data collection. All authors read and approved the final manuscript.

## Funding

The study was funded by the Yunnan Provincial Health Commission, China.

## Conflict of interest

The authors declare that the research was conducted in the absence of any commercial or financial relationships that could be construed as a potential conflict of interest.

## Publisher's note

All claims expressed in this article are solely those of the authors and do not necessarily represent those of their affiliated organizations, or those of the publisher, the editors and the reviewers. Any product that may be evaluated in this article, or claim that may be made by its manufacturer, is not guaranteed or endorsed by the publisher.

## References

[1] GBD 2015 Risk Factors Collaborators. Global, regional, and national comparative risk assessment of 79 behavioural, environmental and occupational, and metabolic risks or clusters of risks, 1990-2015: a systematic analysis for the Global Burden of Disease Study 2015. Lancet. (2016) 388:1659–724. 10.1016/S0140-6736(16)31679-8 27733284 PMC5388856

[2] GBD 2015 Tobacco Collaborators. Smoking prevalence and attributable disease burden in 195 countries and territories, 1990-2015: a systematic analysis from the Global Burden of Disease Study 2015. Lancet. (2017) 389:1885–906. 10.1016/S0140-6736(17)30819-X 28390697 PMC5439023

[3] World Health Organization?. WHO Global Report on Trends in Prevalence of Tobacco Smoking. (2015). Available online at: ttps://apps.who.int/iris/handle/10665/156262 (accessed August 7, 2021).

[4] Ekpu VU Brown AK. The economic impact of smoking and of reducing smoking prevalence: review of evidence. Tob Use Insights. (2015) 8:1–35. 10.4137/TUI.S15628 26242225 PMC4502793

[5] Hu TW Mao Z Ong M Tong E Tao M Jiang H. China at the crossroads: the economics of tobacco and health. Tobacco Control. (2006) 15(Suppl.1):i37–41. 10.1136/tc.2005.014621 16723674 PMC2563551

[6] National Bureau of Statistics of China. National Data. (2021). Available online at: http://data.stats.gov.cn/index.htm (accessed August 7, 2021).

[7] Beijing Intelligence Research Consulting Co. Ltd. 2018–2024 China Tobacco Industry Market In-depth Research Investment Strategy Research Report. (2018). Available online at: http://www.chinairr.org/report/R07/R0701/201806/28-265617.html (accessed August 7, 2021).

[8] Li Y Liu S Wang L Zhou M. Burden of disease attributable to main behavioral risk factor of chronic disease inactivity in China, 1990 and 2010. Chin J Prev Med. (2015) 49:333–8. 10.3760/cma.j.issn.0253-9624.2015.04.012 26081537

[9] Zhou M Wang H Zeng X Yin P Zhu J Chen W. Mortality, morbidity, and risk factors in China and its provinces, 1990–2017: a systematic analysis for the Global Burden of Disease Study 2017. Lancet. (2019) 394:1145–58. 10.1016/S0140-6736(19)30427-1 31248666 PMC6891889

[10] Zhang M Liu S Yang L Jiang Y Wang L. Prevalence of smoking and knowledge about the smoking hazards among 170,000 Chinese adults: a nationally representative survey in 2013–2014. Nicotine Tobacco Res. (2019) 21:10053. 10.1093/ntr/ntz020 30759252

[11] Wang M Luo X Xu S Liu W Ding F Zhang X. Trends in smoking prevalence and implication for chronic diseases in China: serial national cross-sectional surveys from 2003 to 2013. Lancet Respir Med. (2019) 7:35–45. 10.1016/S2213-2600(18)30432-6 30482646

[12] Cai L Wang X Fan L Cui W Golden AR. Socioeconomic disparities in prevalence and behaviors of smoking in rural Southwest China. BMC Public Health. (2019) 19:1117. 10.1186/s12889-019-7455-0 31412820 PMC6694669

[13] Fu SS Burgess D Ryn MV Hatsukami DK Solomon J Joseph AM. Views on smoking cessation methods in ethnic minority communities: a qualitative investigation. Prev Med. (2007) 44:235–40. 10.1016/j.ypmed.2006.11.002 17175016

[14] Wang Q Shen J Sotero M Li C Hou Z. Income, occupation and education: are they related to smoking behaviors in China? PLoS ONE. (2018) 13:e0192571. 10.1371/journal.pone.0192571 29420649 PMC5805321

[15] Cai L Wu XN Goyal A Yuntao H Wenlong C Xia X. Patterns and socioeconomic influences of tobacco exposure in tobacco cultivating rural areas of Yunnan Province, China. BMC Public Health. (2012) 12:842. 10.1186/1471-2458-12-842 23035644 PMC3515419

[16] Chen R Li Y Long W Zhou P Sun T Li F. Survey on tobacco use and associated factors in population in Shandong province, 2016-2017. Chin J Epidemiol. (2021) 42:1200–4. 10.3760/cma.j.cn112338-20200903-01123 34814531

[17] Li Z Yao Y Han W Yu Y Liu Y Tao Y. Smoking prevalence and associated factors as well as attitudes and perceptions towards tobacco control in northeast China. Int J Environ Res Public Health. (2015) 12:8606–18. 10.3390/ijerph120708606 26206569 PMC4515736

[18] National Bureau of Statistics. China Statistical Yearbook 2021. (2022). Available online at: http://www.stats.gov.cn/tjsj/ndsj/2021/indexch.htm (accessed June 16, 2022).

[19] Xu Q Li Y Ma F. The study on accelerating economic development of the Drung nationality in the Gongshan county of Nujiang lisu autonomous prefecture. J Yunnan Agri Univ. (2009) 3:37–54.

[20] Yang G Wang Y Zeng Y Gao GF Liang X Zhou M.. Rapid health transition in China, 1990–2010: findings from the Global Burden of Disease Study 2010. Lancet. (2013) 381:1987–2015. 10.1016/S0140-6736(13)61097-1 23746901 PMC7159289

[21] Jakovljevic M Sugahara T Timofeyev Y Rancic N. Predictors of (in)efficiencies of Healthcare Expenditure Among the Leading Asian Economies – comparison of OECD and Non-OECD Nations. Risk Manag Healthc Policy. (2020) 13:2261–80. 10.2147/RMHP.S266386 33117004 PMC7585857

[22] He P Takeuchi T Yano E. An overview of the China National Tobacco Corporation and State Tobacco Monopoly Administration. Environ Health Prev Med. (2013) 18:85–90. 10.1007/s12199-012-0288-4 22696197 PMC3541807

[23] China Center for Disease Control and Prevention. Report on Chronic Disease Risk Factor Surveillance in China. 1st ed. Beijing: Military Medical Press (2016). p. 60.

[24] Pierce J Messer K White M Cowling D Thomas D. Prevalence of heavy smoking in California and the United States, 1965–2007. J Am Med Assoc. (2011) 305:1106–12. 10.1001/jama.2011.334 21406647

[25] Luo X Duan S Duand Q Pu Y Yang Y Ding Y. Tobacco use among HIV-infected individuals in a rural community in Yunnan Province, China. Drug Alcohol Depend. (2014) 134:144–50. 10.1016/j.drugalcdep.2013.09.023 24144787

[26] Luo X Duan S Duand Q Pu Y Yang Y Wong F. Prevalence and correlates of alcohol use and subsequent sexual activity among adult males in a rural community of ethnic minorities in Yunnan Province, China. Biosci Trends. (2012) 6:288–95. 10.5582/bst.2012.v6.6.288 23337788

[27] World Health Organization. International Guide for Monitoring Alcohol Consumption and Related Harm. Geneva: World Health Organization (2000). p. 54.

[28] Vandendijck Y Faes C Hens N. Prevalence and trend estimation from observational data with highly variable post-stratification weights. Ann Appl Stat. (2016) 10:94–117. 10.1214/15-AOAS874

[29] Henriksen L. Comprehensive tobacco marketing restrictions: promotion, packaging, price and place. Tob Control. (2012) 21:147–53. 10.1136/tobaccocontrol-2011-050416 22345238 PMC4256379

[30] Li S Ma S Xi B. Tobacco control in China: still a long way to go. Lancet. (2016) 387:1375–6. 10.1016/S0140-6736(16)30080-0 27115816

[31] Wang JF Ma H Wang ZZ Zhang YH. Prevalence of tabacco and alcohol use in ethnic Hui and Han residents in Ningxia. Chin J Epidemiol. (2015) 36:1231–5. 26850242

[32] Li WQ. Association Between Smoke and Carotid Intima-Media Thickness in Xinjiang Adult Population. (Doctoral thesis), Xinjiang Medical University, Urumqi, China (2011).

[33] Cao F Cai L Cui WL Sun CH He JQ. Socioeconomic difference in prevalence of nicotine dependence among Naxi ethnic minority in Yulong County of Yunnan Province. Chin J Dis Control Prev. (2016) 20:452–5. 10.16462/j.cnki.zhjbkz.2016.05.006

[34] Wu BQ Gao YD Yang RC Yang JX Lin YK Gou CC. Comparative study of differences in behavior and awareness related to smoking among ethnic groups in Dehong. Yunnan Province Modern Prev Med. (2016) 43:2785–9.

[35] Jakovljevic M Jakab M Gerdtham U McDaid D Ogura S Varavikova E. Comparative financing analysis and political economy of non-communicable diseases. J Med Econ. (2019) 22:722–7. 10.1080/13696998.2019.1600523 30913928

[36] Shao Y Yang Y Xiao Y Xu W Tang X Shi Q. Cross-sectional survey on smoking in adults of Yunnan province in 2013. China J Front Medical Sci. (2017) 9:28–33. 10.12037/YXYQY.2017.12-07

[37] Zhang M Wang L Li Y Li X Jiang Y Hu N. Cross-sectional survey on smoking and smoking cessation behaviors among Chinese adults in 2010. Chin J Prev Med. (2012) 46:404–8. 10.3760/cma.j.issn.0253-9624.2012.05.006 22883725

[38] Ma G Kong L Luan D Li Y Hu X Wang J. The descriptive analysis of the smoking pattern of people in China. Chin J Prev Contr Chron Non-commun Dis. (2005) 13:195–9. 10.3969/j.issn.1004-6194.2005.05.002

[39] He H Pan L Cui Z Sun J Shan G. Smoking prevalence, patterns, and cessation among adults in Hebei province, central China: implications from China National Health Survey (CNHS). Front Public Health. (2020) 8:1–13. 10.3389/fpubh.2020.00177 32596196 PMC7300263

[40] Fu H Feng D Tang S He Z Xiang Y Wu T. Prevalence of tobacco smoking and determinants of success in quitting smoking among patients with chronic diseases: a cross-sectional study in rural Western China. Int J Environ Res Public Health. (2017) 14:167. 10.3390/ijerph14020167 28208782 PMC5334721

[41] Fang Q Liu Z Li Z Liu Y Zhang Z Zhang Y. Investigation on attitude toward smoking control and practice of suggesting patients giving up smoking among doctors in two cities of Sichuan. Chin J Health Educ. (2009) 25:723–9. 10.16168/j.cnki.issn.1002-9982.2009.10.001

[42] Honjo K Iso H Inoue M Tsugane S Study Group JPHC. Smoking cessation: predictive factors among middle-aged Japanese. Nicotine Tob Res. (2010) 12:1050–4. 10.1093/ntr/ntq143 20819787

[43] Hagen E Garfield M Sullivan R. The low prevalence of female smoking in the developing world: gender inequality or maternal adaptations for fetal protection? Evol Med Public Health. (2016) 2016:195–211. 10.1093/emph/eow013 27193200 PMC4931906

[44] Peto R Chen Z Boreham J. Tobacco: the growing epidemic in China. CVD Prev Control. (2009) 4:61–70. 10.1016/j.cvdpc.2008.12.001

[45] Yuan Z. Sociological analysis of minority females' health and smoking in Yunnan Frontier. J Kunming Univ. (2008) 19:16–20.

[46] Benard A Bonnet F Tessier J Fossoux H Dupon M Mercie P. Tobacco addiction and HIV infection: toward the implementation of cessation programs. ANRS CO_3_ Aquitaine cohort. AIDS Patient CareSTDS. (2007) 21:458–68. 10.1089/apc.2006.0142 17651027

[47] Centers for Disease Control and prevention. Cigarette smoking among adults and trends in smoking cessation - United States 2008. Morb Mortal Wkly Rep. (2009) 58:1227–32. 19910909

[48] Cox LS Okuyemi K Choi WS Ahluwalia JS. A review of tobacco use treatments in US. Ethnic minority populations. Am J Health Promot. (2011) 25:S11–30. 10.4278/ajhp.100610-LIT-177 21510783 PMC4494679

[49] Xiao L Jiang Y Liu X Li Y Gan Q Liu F. Smoking reduced in urban restaurants: the effect of Beijing Smoking Control Regulation. Tob Control. (2017) 26:e75–8. 10.1136/tobaccocontrol-2016-053026 27413062

[50] Yang G Wang Y Wu Y Yang J Wan X. The road to effective tobacco control in China. Lancet. (2015) 385:1019–28. 10.1016/S0140-6736(15)60174-X 25784349

[51] Redmon P Koplan J Eriksen M Li S Kean W. The role of cities in reducing smoking in China. Int J Environ Res Public Health. (2014) 11:10062–75. 10.3390/ijerph111010062 25264682 PMC4210967

[52] Tan X Liu X Shao H. Healthy China 2030: A vision for health care. Value Health Regional Iss. (2017) 12:112–4. 10.1016/j.vhri.2017.04.001 28648308

