# Supplementary material for: Prevalence and correlates of cigarette smoking among Dulong adults in China: A cross-sectional survey in 2020
Source: Front Public Health. 2022 Oct 13;10:973583. doi: 10.3389/fpubh.2022.973583 (PMC9608327; doi:10.3389/fpubh.2022.973583)
Supplement: Supplementary file 1 [file Presentation_1.pdf]

云南省疾病预防控制中心科学研究伦理委员会

伦理审查批件

批件号	伦审批 2020-11 号		
项目名称	云南省独龙族居民健康状况调查与评估		
项目来源	云南省卫健委基层处		
研究单位与部门	慢性非传染病防制所		
主要研究者	秦明芳		
审查方式	会议审查	审查日期	2020.6.22
审查委员	查舜、代解杰、丁峥嵘、李瑛、林丽、王荣华、高莉、杨祖顺		
审查文件	1、　知情同意书；2、研究方案及相关资料；3、主要研究者简历；		
审查意见	同意		
批件有效期	2020 年 6 月 23 日-2020 年 12 月 31 日		
主任委员签字	查舜		
伦理委员会（盖章）	云南省疾病预防控制中心科学研究伦理委员会		
日期	2020 年 6 月 23 日		
