# Supplementary material for: Prevalence and correlates of cigarette smoking among Dulong adults in China: A cross-sectional survey in 2020
Source: Front Public Health. 2022 Oct 13;10:973583. doi: 10.3389/fpubh.2022.973583 (PMC9608327; doi:10.3389/fpubh.2022.973583)
Supplement: Supplementary file 2 [file Presentation_2.pdf]

Scientific Research Ethics Committee, Yunnan Center for Disease Control and Prevention

Ethics Review Approval

**The certificate NO.**	Lunshenpi-2020-11		
**The project name**	Dulong Health Status Investigation and Evaluation		
**Project source**	Yunnan Provincial Health Commission		
**Research units and departments**	Division for prevention and control of chronic non-communicable disease，Yunnan center for disease control and prevention		
**Principal investigator**	Mingfang Qin		
**Review method**	Meeting review	**Review date**	22 June 2020
**Review committee**	Zha Shun, Dai Jiejie, Ding Zhengrong, Li Ying, Lin Li, Wang Ronghua, Gao Li, Yang Zushun		
**Review documents**	1. Informed consent; 2. Research proposal and related materials; 3. The main investigator's resume;		
**Review comments**	Reviewed and approved		
**Validity of approval**	23 June 2020-31 December 2020		
**Signature of Chairman**	（View in original）		
**Seal of ethics Committee**	（View in original）		
**Approval date**	23 June 2020		
